# Subclinical myocardial inflammation and diffuse fibrosis are common in systemic sclerosis – a clinical study using myocardial T1-mapping and extracellular volume quantification

**DOI:** 10.1186/1532-429X-16-21

**Published:** 2014-03-04

**Authors:** Ntobeko AB Ntusi, Stefan K Piechnik, Jane M Francis, Vanessa M Ferreira, Aitzaz BS Rai, Paul M Matthews, Matthew D Robson, James Moon, Paul B Wordsworth, Stefan Neubauer, Theodoros D Karamitsos

**Affiliations:** 1 Radcliffe Department of Medicine, Division of Cardiovascular Medicine, Level 0, John Radcliffe Hospital, University of Oxford Centre for Clinical Magnetic Resonance Research, Oxford OX3 9DU, United Kingdom; 2 GlaxoSmithKline Clinical Imaging Centre, London, UK; 3 Division of Brain Sciences, Department of Medicine, Imperial College, London, UK; 4 Institute of Cardiovascular Science, University College London & Heart Hospital, London, UK; 5 Nuffield Department of Orthopaedics & NIHR Oxford Musculoskeletal Biomedical Research Unit, Rheumatology and Musculoskeletal Sciences, University of Oxford, Nuffield Orthopaedic Centre and John Radcliffe Hospital, Oxford, UK

**Keywords:** Scleroderma, Cardiovascular magnetic resonance, T1 mapping, Extracellular volume estimation, Gadolinium

## Abstract

**Background:**

Systemic sclerosis (SSc) is characterised by multi-organ tissue fibrosis including the myocardium. Diffuse myocardial fibrosis can be detected non-invasively by T1 and extracellular volume (ECV) quantification, while focal myocardial inflammation and fibrosis may be detected by T2-weighted and late gadolinium enhancement (LGE), respectively, using cardiovascular magnetic resonance (CMR). We hypothesised that multiparametric CMR can detect subclinical myocardial involvement in patients with SSc.

**Methods:**

19 SSc patients (18 female, mean age 55 ± 10 years) and 20 controls (19 female, mean age 56 ± 8 years) without overt cardiovascular disease underwent CMR at 1.5T, including cine, tagging, T1-mapping, T2-weighted, LGE imaging and ECV quantification.

**Results:**

Focal fibrosis on LGE was found in 10 SSc patients (53%) but none of controls. SSc patients also had areas of myocardial oedema on T2-weighted imaging (median 13 vs. 0% in controls). SSc patients had significantly higher native myocardial T1 values (1007 ± 29 vs. 958 ± 20 ms, p < 0.001), larger areas of myocardial involvement by native T1 >990 ms (median 52 vs. 3% in controls) and expansion of ECV (35.4 ± 4.8 vs. 27.6 ± 2.5%, p < 0.001), likely representing a combination of low-grade inflammation and diffuse myocardial fibrosis. Regardless of any regional fibrosis, native T1 and ECV were significantly elevated in SSc and correlated with disease activity and severity. Although biventricular size and global function were preserved, there was impairment in the peak systolic circumferential strain (-16.8 ± 1.6 vs. -18.6 ± 1.0, p < 0.001) and peak diastolic strain rate (83 ± 26 vs. 114 ± 16 s-1, p < 0.001) in SSc, which inversely correlated with diffuse myocardial fibrosis indices.

**Conclusions:**

Cardiac involvement is common in SSc even in the absence of cardiac symptoms, and includes chronic myocardial inflammation as well as focal and diffuse myocardial fibrosis. Myocardial abnormalities detected on CMR were associated with impaired strain parameters, as well as disease activity and severity in SSc patients. CMR may be useful in future in the study of treatments aimed at preventing or reducing adverse myocardial processes in SSc.

## Background

Systemic sclerosis (SSc) is an autoimmune connective tissue disorder characterised by vascular dysfunction and multi-organ fibrosis. The heart is one of the major organs commonly involved in SSc, with an estimated clinical prevalence of 15-35% [1]. Cardiovascular disease (CVD) in SSc may be direct (cardiac fibrosis, myocarditis, dilated cardiomyopathy, cardiac failure, premature coronary artery disease, conduction system abnormalities, valvular disease and pericardial disease) or indirect (pulmonary hypertension and renal crisis) [2,3]. In the majority of SSc patients, however, CVD often remains subclinical [4]. SSc patients with apparent cardiovascular clinical features are at greater risk of deterioration and premature cardiovascular death [5]. Therefore, early detection and monitoring of myocardial and vascular involvement is a crucial aspect of management [6].

Diffuse myocardial fibrosis is the pathological hallmark of cardiovascular involvement in SSc, reported in up to 80% of cases in autopsy studies [7], and is thought to represent the final common expression of ‘contraction band necrosis’, recurrent episodes of ischaemia-reperfusion injury, microvascular dysfunction and chronic myocardial inflammation [4,8,9]. However, myocardial inflammation and diffuse fibrosis are difficult to detect clinically, and endomyocardial biopsy is limited by sampling error, low diagnostic sensitivity and its invasive nature [10].

Cardiovascular magnetic resonance (CMR) can non-invasively detect myocardial inflammation and fibrosis. T2-weighted imaging can identify areas of myocardial oedema, and late gadolinium enhancement (LGE) imaging can identify areas of focal fibrosis in patients with SSc [11,12]. However, conventional T2-weighted imaging only has modest sensitivity in detecting myocardial oedema [13,14], especially in mild cases, and LGE is suboptimal as a technique to detect diffuse myocardial fibrosis [15,16]. Recently, T1 mapping and extracellular volume (ECV) quantification have emerged as novel methods that can overcome both of these limitations and are promising to detect subtle forms of myocardial inflammation and diffuse myocardial fibrosis. T1 mapping is highly sensitive to myocardial water and is superior to T2-weighted imaging in detecting myocardial oedema [14,17]. T1 mapping and ECV measurements can also act as surrogates for diffuse fibrosis [18,19] on the premise of detecting myocardial water in the expanded interstitial space, and have been shown to correlate well with histological indices of myocardial fibrosis in various clinical contexts [20,21].

We therefore hypothesised that T1 and ECV quantification would reveal subclinical myocardial involvement in asymptomatic SSc patients with no known cardiovascular involvement when compared to controls of similar age and sex. We also sought to investigate the relationship between myocardial findings on CMR and indices of SSc activity, severity and chronicity, and early signs of myocardial dysfunction.

## Methods

### Study population

This was a prospective study enrolling unselected patients with SSc (n = 19), without any known CVD. Patients were recruited from 5 hospitals in the Thames Valley, United Kingdom (John Radcliffe Hospital, Oxford; Nuffield Orthopaedic Centre, Oxford; Great Western Hospital, Swindon; Royal Berkshire Hospital, Reading; and Stoke Mandeville Hospital, Aylesbury) between January 2011 and December 2012. The SSc patients were between the ages of 18 and 65 years, and were diagnosed with SSc using the 1980 American College of Rheumatology criteria [22]. Exclusion criteria included inability to tolerate CMR, contraindications to CMR, non-sinus rhythm, known heart disease (previous myocardial infarction, previous myocarditis, heart failure, arrhythmia on 12-lead ECG and medical history or other chronic cardiac condition), renal impairment (estimated glomerular filtration rate below 30 mL/min), impaired liver function (alanine aminotransferase greater than twice the upper limit of normal), a female who was pregnant, lactating or planning a pregnancy, and known hypersensitivity to gadolinium. Age- and sex-matched healthy individuals (n = 20) with no cardiac history, not on cardiovascular medications (except 3 on hormone replacement therapy) and with a normal ECG were used for comparison. All subjects gave written informed consent to participate in the study. Ethical approval was granted for all study procedures by the Oxford Research Ethics Committee (REC Ref 10/H0606/32).

### CMR

CMR studies were performed using a single 1.5 T MR system (Avanto, Siemens Healthcare, Germany). A 32-channel phased-array chest coil was used for all data acquisition, except for STIR imaging, for which the body coil was used. A complete stack of short axis images were obtained during breath hold and cardiac gating for cine, precontrast (native) T1 mapping, T2-weighted and LGE imaging. T1 mapping was performed using the ShMOLLI (Shortened Modified Look-Locker Inversion Recovery) sequence [19], and T2 weighted-CMR was performed with the black blood short-Tau inversion recovery (STIR) sequence as previously published [23]. Tagged cine CMR was acquired with an ECG-triggered segmented k-space gradient echo sequence with spatial modulation of magnetisation (SPAMM) in orthogonal planes [24]. Three short axis (basal, mid-ventricular and apical) scans and a single long axis (horizontal) scan were obtained for tagging. T2-weighted and cine tagged images were acquired before administration of contrast agent. LGE imaging was performed as previously described [25], using a T1-weighted phase-sensitive inversion recovery sequence about 8 minutes after intravenous administration of contrast agent (Gadoterate meglumine–Gd-DOTA, Dotarem, Guerbet LLC, France; 0.15 mmol/kg body weight). A single mid-ventricular short axis slice was acquired for postcontrast T1 maps at 1, 2, 3, 4, 8, 15 and 20 minutes after the administration of contrast (Gd-DOTA). Typical imaging parameters for the sequences used were as previously published [14].

### CMR image analysis

All CMR images and maps were analysed offline in a blinded fashion.

#### Cine images

Analysis of left ventricular ejection fraction was performed using Argus software (Version VB17, 2011, Siemens Medical Solutions). Left ventricular (LV) short axis epicardial and endocardial borders were manually contoured at end-diastole and end-systole. LV end systolic (LVESV) and end diastolic (LVEDV) volumes were used to calculate stroke volume (SV) and ejection fraction (EF) – (EF = SV/EDV). Myocardial mass was also calculated by subtracting the endocardial volume from the epicardial volume, based on prior knowledge of myocardial specific gravity (1.05 g/cm^3^). Left atrial diameter was measured in the LV outflow tract (3-chamber) view.

#### Tagged cine images

Post-processing and semi-automated analysis was performed using CIM software (CIMTag2D, Auckland, New Zealand) by aligning a grid to the myocardial tagging planes in end-diastole. End-systole was determined visually, and tags are adjusted at each frame through the cardiac cycle. From the mid-short axis slice, peak circumferential systolic strain and peak diastolic strain rate were derived.

### STIR images

Quantitative analysis was performed by comparing the LV myocardium in short axis against adjacent skeletal muscle in the same slice, verified on a corresponding SSFP image. The T2 signal intensity (SI) ratio was calculated as T2 SI_myocardium:skeletal_ = SI_myocardium_/SI_skeletal__muscle_, as previously published [23]. Myocardial oedema was diagnosed when myocardial T2 SI ratio is > 1.9. Care was taken to exclude non-suppressed blood pool signal due to slow-flow adjacent to the subendocardium and to avoid using areas with abnormally low signal for normalisation.

#### LGE images

Images were evaluated qualitatively for the presence or absence, pattern (subendocardial, midwall, subepicardial, transmural) and regional distribution of LGE areas by three observers, each with at least 4 years of CMR experience. The detection of LGE was made by consensus of all 3 observers. In addition, endocardial and epicardial regions of interest (ROI) were manually contoured in the LGE images, together with a reference ROI in the anterior LV wall without visual LGE, and focal areas of LGE were defined quantitatively as those with SI ≥ 2.0 standard deviations above the mean SI of normal myocardium.

#### T1-maps

Analysis of T1 mapping was performed as previously described [19]. Briefly, after T1maps were generated, short axis images were manually contoured using in-house software MC-ROI (programmed by SKP in Interactive Data Language, version 6.1, Exelis Visual Information Solutions, Boulder, Colorado, USA) to outline the endo- and epicardium, and then divided into 6 segments per slice using the anterior right ventricular RV-LV insertion point as reference and for comparing segments amongst sequences [14].

Consistent with established methods of estimating myocardial ECV using a delayed postcontrast bolus protocol [26], we measured precontrast and postcontrast myocardial and blood T1 values. The estimation of ECV and lambda (λ) was based on multipoint regression [27], incorporating all available precontrast and postcontrast points, in order to increase the robustness of the estimates by increasing number of underlying data points. ECV was calculated as (1 – haematocrit). We have not observed any consistent deviations from the linear regression line in our data (median R = 0.99, interquartile range = 0.99 to 1.00) to support effects of transcytolemmal exchange, indicating that fast exchange effects only contribute less that 0.1% effect on ECV [28]. We also checked that the estimates obtained from the latest postcontrast time (~20 minutes) did not yield any significant difference both across all (P > 0.9) and in the most affected subgroup (P = 0.8) to exclude any potential effects of sub-equilibrium gadolinium redistribution. For calculation of postcontrast T1 values, the postcontrast T1 map acquired at 20 minutes was utilised.

#### Areas of myocardial involvement by STIR and precontrast T1 mapping

Briefly, on dark-blood T2W images, oedema was diagnosed when myocardial T2 SI is ≥ 1.9 compared to that of skeletal muscle [13]; on T1 maps, acute myocardial injury was diagnosed when T1 was > 990 ms, as previously published for the objective detection of acute myocardial oedema [17]. For all quantitative analyses of T2-weighted and T1 map images, only regions of myocardium with a contiguous area of ≥40 mm^2^ above the specified thresholds were considered relevant. This corresponds to 10 adjacent pixels for the STIR method, in accordance with currently proposed recommendations [13], to reduce the detection of noise as positive findings. To calculate the extent of myocardial involvement in a subject detected by the tissue characterisation techniques, the percentage of abnormal myocardium as defined above was determined for each segment and then averaged for that subject.

### Echocardiography

Two-dimensional, M-mode and Doppler echocardiograms were acquired using a Toshiba Artida 4D system (Toshiba Medical Systems Corporation, Tokyo, Japan). Images were acquired with the patients in the left lateral decubitus position. Primary measurements of mitral inflow included the peak early filling (E-wave) and late diastolic filling (A-wave) velocities, the E/A ratio, deceleration time (DT) of early filling velocity, which were derived by placing the cursor of the pulsed wave Doppler in the LV, above the tips of the mitral valve, to display the onset of mitral inflow, using a 5 MHz transducer. The passive LV filling (E’-wave) was measured from the pulsed wave tissue Doppler of the mitral septal annular velocity. Right ventricular systolic pressure was based on measurement of maximal tricuspid regurgitation velocity and applying the modified Bernoulli equation before addition of the estimated right atrial pressure (5 mmHg).

### SSc disease activity and severity

Disease activity was assessed using the Valentini disease activity index (VDAI) of the European Scleroderma Study Group criteria for disease activity in SSc [29], which incorporates skin changes, digital necrosis, lung function tests, ESR and serum complement.

SSc patients were classified as having either diffuse (n = 10) or limited cutaneous SSc (n = 9). The severity of skin fibrosis was quantified using the modified Rodnan skin score (mRSS) [30], a measure of SSc disease severity and activity based on skin thickness at 17 anatomical sites.

### Statistical analysis

Normality of data was tested using the Kolmogorov-Smirnov test. Normally distributed data are presented as mean ± standard deviation (SD) or, where highly skewed, as median (interquartile range); non-parametric data are presented as numbers (percentages). The chi-square test or Fischer’s exact test was used to compare dichotomous data. The unpaired Student t-test (when normally distributed) or Mann-Whitney U test (for non-parametric data) was used to compare continuous variables between SSc patients and controls, as appropriate. Post-hoc Bonferroni correction was used to explore whether there were differences between the SSc patients and controls. Any segmental data were averaged on a per-subject basis before group comparisons to control for clustering of segments within each subject. Bivariate correlations were assessed using the Pearson R or Spearman R_S_ coefficient, as appropriate. All statistical tests were two-tailed and a p-value of less than 0.05 was considered statistically significant. All analysis was performed using SPSS version 20 (IBM, Armonk, New York, USA).

## Results

### Baseline characteristics of the patient population

The SSc patients were well-matched with controls for age, sex and comorbidities and only a small minority of patients were on regular disease modifying anti-rheumatic drugs (Table 1). Most patients had been diagnosed with SSc for more than a decade (median disease duration 14 years, IQR 5-19). In SSc patients, the VDAI and the mRSS were 4 ± 2 and 20 ± 6, respectively, indicating the presence of active disease and organ involvement in the group overall.

**Table 1:** Baseline characteristics of the study population

	**Controls**	**SSc**	**P value**
	**N = 20**	**N = 19**	
Demographic and clinical features and co-morbidity			
Female sex, n (%)	19 (95)	18 (95)	0.74
Age, years	56 ± 8	55 ± 10	0.64
Current smokers, n (%)	0 (0)	2 (11)	-
Hypertension, n (%)	2 (10)	4 (21)	0.41
Diabetes, n (%)	0 (0)	0 (0)	–
Hyperlipidaemia, n (%)	4 (20)	3 (16)	0.73
Obesity, n (%)	2 (10)	4 (21)	0.34
BMI, kg/m^2^	25 ± 4	27 ± 7	0.23
Medical therapy			
Methotrexate, n (%)	None	5 (26)	–
Prednisolone, n (%)	None	2 (11)	–
Azathioprine, n (%)	None	1 (5)	–
Chloroquine, n (%)	None	1 (5)	–
Leflunomide, n (%)	None	1 (5)	–
Sulfasalazine, n (%)	None	0 (0)	–
HRT, n (%)	3 (15)	4 (21)	0.62
NSAID, n (%)	None	3 (16)	–
Duration of DMARDs, years (median, IQR)	N/A	2 (1-8)	–
Duration of NSAIDs, years (median, IQR)	N/A	1 (1-4)	–
Disease activity and chronicity indices			
SSc VDAI	N/A	4 ± 2	–
ESR, mm/hr (median, IQR)	N/A	11 (3-18)	–
CRP, mg/L (median, IQR)	3 (1-4)	5 (2-8)	0.01
Hemoglobin (g/L)	13 ± 1	12 ± 1	0.05
Haematocrit (%)	41 ± 11	34 ± 9	0.001
Creatinine (μmol/L)	N/A	67 ± 10	–
Duration of SSc, years (median, IRQ)	N/A	14 (5-19)	–
mRSS	N/A	20 ± 6	–
Limited/diffuse cutaneous SSc, n (%)	N/A	10/9	–
Anti-centromere antibodies, n (%)	N/A	8 (42)	–
Anti-topoisomerase 1 antibodies, n (%)	N/A	5 (26)	–

Continuous data are mean ± SD unless otherwise indicated.

*AID*, Autoimmune disease; *BMI*, Body mass index; *CAD*, Coronary artery disease; *CRP*, C-reactive protein; *DMARD*, Disease modifying anti-rheumatic drug; *ESR*, Erythrocyte sedimentation rate; *HRT*, Hormone replacement therapy; *mRSS*, Modified Rodnan skin score; *NSAID*, Non-steroidal anti-inflammatory drug; *SSc*, Systemic sclerosis; *VDAI*, Valentini disease activity index of the European Scleroderma Study Group.

### Myocardial structure and function

There was no significant difference in LV size, mass and ejection fraction between SSc patients and controls (Table 2). Despite normal global LV systolic function on cine imaging, peak systolic circumferential strain by tagged CMR was impaired in SSc patients compared to controls (-16.8 ± 1.6 vs. -18.6 ± 1.0, p < 0.001), indicating an abnormality in regional function and myocardial deformation characteristics. The left atrial (LA) diameter was larger in SSc patients (37 ± 6 vs. 28 ± 5 mm, p < 0.001), likely due to diastolic dysfunction, as demonstrated by the reduced peak diastolic strain rate in SSc patients (83 ± 26 vs. 114 ± 16 s^-1^, p < 0.001) compared to controls. Evidence of impaired diastolic function was also confirmed on echocardiographic assessment, which showed abnormal relaxation indices in SSc patients (E/A: 1.8 ± 0.5 vs. 1.4 ± 0.2, p = 0.039; and E/E’: 11 ± 4 vs. 7 ± 1, p = 0.006).

**Table 2:** CMR findings

	**Controls**	**SSc**	**P value**
	**N = 20**	**N = 19**	
LVEDV indexed, mL/m^2^	77 ± 16	69 ± 11	0.08
LVESV indexed, mL/m^2^	21 ± 5	18 ± 5	0.06
LVEF, %	73 ± 5	74 ± 6	0.52
LV Mass indexed, g/m^2^	52 ± 11	51 ± 8	0.74
LA size, mm	28 ± 5	37 ± 6	<0.001
RVEDV indexed, mL/m^2^	85 ± 19	77 ± 12	0.32
RVESV indexed, mL/m^2^	28 ± 7	25 ± 7	0.06
RVEF, %	67 ± 4	67 ± 6	0.14
Mid SA circumferential strain	-18.6 ± 1.0	-16.8 ± 1.6	<0.001
Peak diastolic circumferential strain rate (s-1)	114 ± 16	83 ± 26	<0.001
Presence of LGE (%)	0	10 (53)	–
Volume fraction of LGE > 2SD (%)	0	4 (2-5)	–
Global myocardial T2 SI Ratio	1.6 ± 0.5	1.7 ± 0.4	0.66
Volume fraction of oedema by T2 (%)	0	13 (0-21)	–
Average myocardial T1, ms	958 ± 20	1007 ± 29	<0.001
Volume fraction of T1 > 990 ms (%)	3 (1-9)	52 (40-70)	<0.001
Postcontrast T1, ms	454 ± 29	457 ± 50	0.9
λ	0.44 ± 0.15	0.56 ± 0.23	0.02
ECV (%)	27.6 ± 2.5	35.4 ± 4.8	<0.001

Continuous data are mean ± SD unless otherwise indicated.

*LA*, Left atrium; *LGE*, Late gadolinium enhancement; *LV*, Left ventricle/ventricular; *LVEDV*, Left ventricular end-diastolic volume; *LVEF*, Left ventricular ejection fraction; *LVESV*, Left ventricular end-systolic volume; *RVEDV*; Right ventricular end-diastolic volume; *RVEF*, Right ventricular ejection fraction; *RVESD*, Right ventricular end-systolic volume; *SA*, Short axis; *SSc*, Systemic sclerosis; *STIR*, Short Tau inversion recovery.

For postcontrast T1, λ and ECV, the number of SSc patients included in the analysis is 14 rather than 19.

We found no difference in RV size and global systolic function between SSc patients and controls. While right ventricular systolic pressures were higher in the SSc patients (24 ± 5 vs. 16 ± 4 mmHg, p < 0.001), they were within normal limits for all study subjects, excluding the presence of significant pulmonary hypertension in this cohort.

### Patchy fibrosis (LGE imaging)

**Figure 1:**
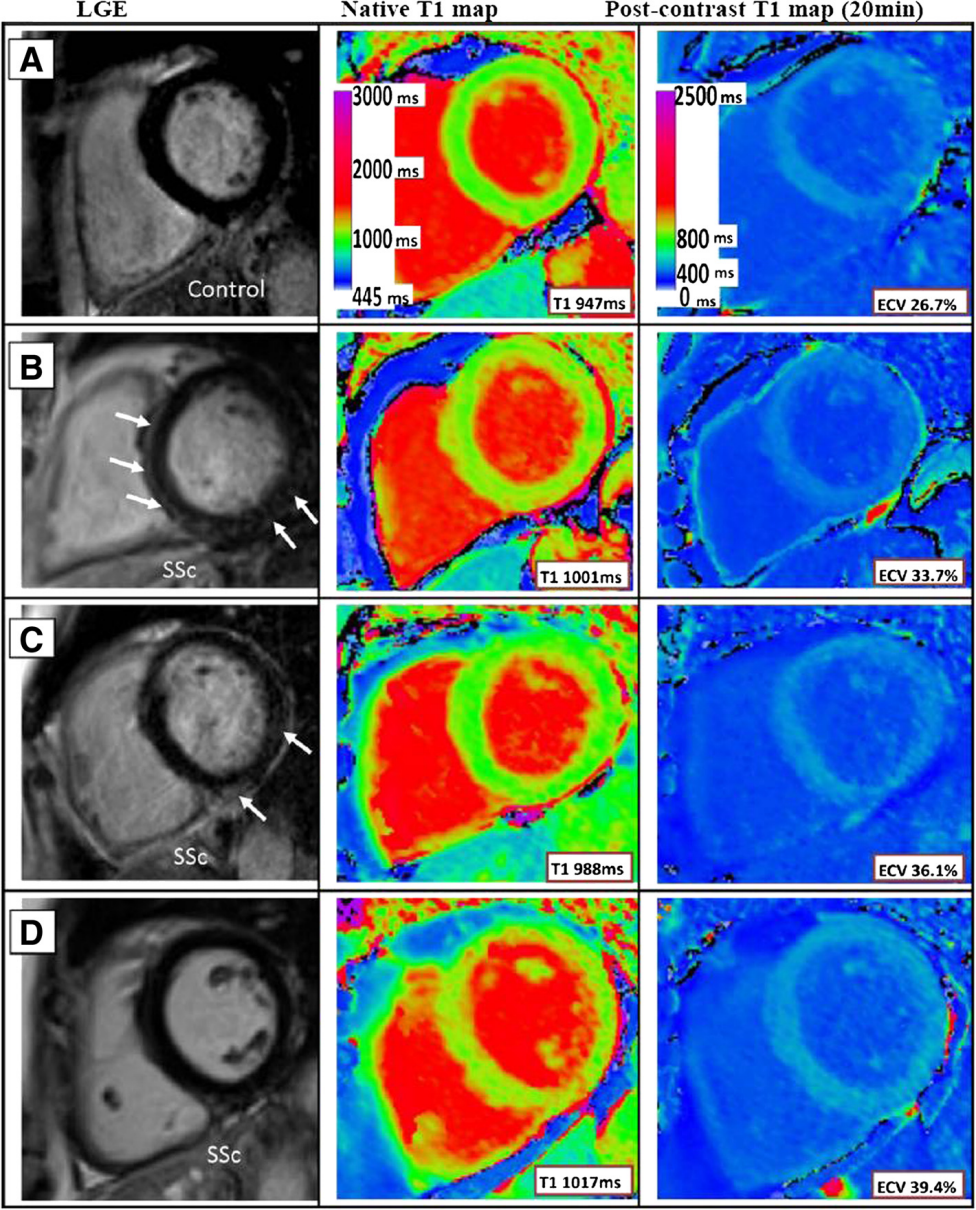
**Representative examples of pre- and postcontrast T1 maps with corresponding LGE images in SSc and controls.** Top panel **(A)**: normal control with no LGE, native T1 947 ms, postcontrast T1 514 ms, ECV 26.7%; Second panel **(B)**: SSc patient with linear septal and patchy basal inferolateral LGE, native T1 1001 ms; postcontrast T1 453 ms; ECV 33.7%; Third panel **(C)**: SSc patient with small areas of mid-wall inferior and lateral LGE, native T1 988 ms, postcontrast T1 439 ms, ECV 36.1%; Fourth panel **(D)**: SSc patient with no LGE, native T1 1017 ms, postcontrast T1 421 ms, ECV 39.4%. LGE, late gadolinium enhancement. Note the scale change between precontrast and postcontrast T1 maps.

Confirming previously published data, we found increased incidence of LGE in SSc patients compared to matched controls (53% vs. 0%), as shown in Table 2. All had a non-ischemic pattern of fibrosis, with about a third of SSc patients demonstrating patchy mid-wall LGE in the basal inferolateral wall; and 21% in the septum (Figure 1). No patient had any previous myocardial infarction (isolated LGE involving the subendocardium). Overall, SSc patients had a small volume of scarring by LGE (3.8 ± 0.4% of total LV mass).

### Myocardial oedema (T2-weighted CMR)

On conventional dark-blood T2-weighted imaging, while there was no significant difference in the overall global myocardial T2 SI ratio in SSc patients compared to controls (1.7 ± 0.4 vs. 1.6 ± 0.5, p = 0.66), SSc patients had significantly more areas of focal myocardial oedema within the left ventricle (median 13% vs. 0% in controls; p < 0.001).

### T1 mapping and extracellular volume (ECV) quantification

**Figure 2:**
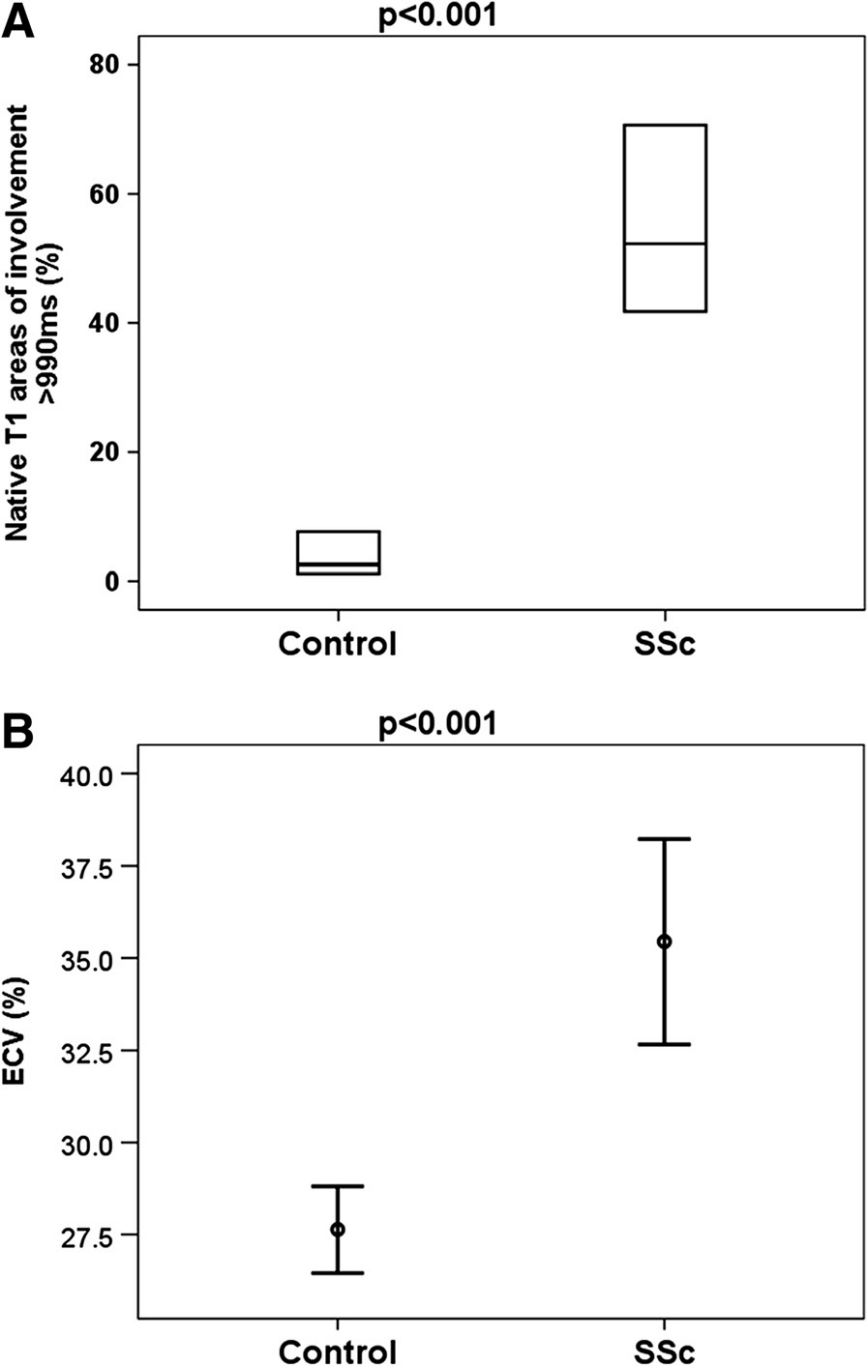
**Myocardial T1 and extracellular volume (ECV) quantification in SSc patients and normal controls.** ECV, extracellular volume, SSc, systemic sclerosis. **A**, Median T1 area >990 ms in SSc and controls (horizontal line indicates median, box indicates 25^th^ and 75^th^ centiles); **B**, Mean ECV values in SSc and controls (error bars indicate 95% confidence interval).

Native T1 mapping was performed in all SSc patients; and postcontrast T1 mapping was done in 14 of the 19 SSC patients, as 5 patients were not consented for gadolinium. SSc patients had significantly higher average myocardial T1 values (1007 ± 29 vs. 958 ± 20 ms, p < 0.001) and larger areas of myocardial involvement detected by native T1 mapping (median 52% vs. 3% in controls, p < 0.001). SSc patients also had significantly higher λ (0.56 ± 0.23 vs. 0.44 ± 0.15, p = 0.02) and expanded ECV (35.4 ± 4.8 vs. 27.6 ± 2.5%, p < 0.001; Figure 2).

Postcontrast T1 and λ values were found to be less good as measures of diffuse myocardial fibrosis. There was no difference in postcontrast T1 values between SSc patients and controls (T1 at 20 min post gadolinium: 457 ± 50 vs. 454 ± 29 ms, p = 0.9). Also, there was no difference in postcontrast T1 values between controls and SSc patients with LGE, despite the significant differences in native T1 and ECV. Importantly, between SSc patients without LGE and SSc patients with LGE, there was no difference in native T1, postcontrast T1, λ, and ECV, indicating that the increase in ECV was not driven by the presence of LGE (Table 3).

**Table 3:** Measures of subclinical myocardial involvement by T1 mapping and ECV in SSc patients with and without LGE

	**SSc without LGE**	**SSc with LGE**	**P value**
	**N = 9**	**N = 10**	
Volume fraction of LGE > 2SD (%)	0	6 (3-9)	–
Precontrast T1 (ms)	1007 ± 36	1006 ± 17	0.99
Volume fraction of T1 > 990 ms (%)	55 (48-78)	51 (39-73)	0.78
Postcontrast T1 (ms)*	470 ± 69	443 ± 12	0.57
λ	0.52 ± 0.21	0.62 ± 0.23	0.13
ECV (%)	34 ± 4	37 ± 5	0.18
STIR T2 Ratio	1.7 ± 0.4	1.6 ± 0.3	0.65
Volume fraction of oedema (T2 STIR SI >1.9, %)	21 (9-23)	14 (6-17)	0.55

Data are mean ± SD unless otherwise indicated.

*At 20 minutes post gadolinium administration.

λ, Myocardial partition coefficient (Lambda); *ECV*, Extracellular myocardial volume; *LGE*, Late gadolinium enhancement; *STIR*, Short Tau inversion recovery.

For postcontrast T1, λ and ECV, the number of SSc patients included in the analysis is 14 rather than 19.

When SSc patients were stratified according to limited cutaneous or diffuse SSc, only native T1 mapping and ECV quantification were able to further differentiate the two subgroups according to myocardial involvement (Table 4): patients with diffuse SSc had higher native T1 values (1011 ± 24 vs. 1002 ± 32 ms, p = 0.01), larger areas of myocardial involvement by native T1 mapping (median 63% vs. 45%, p < 0.001) and larger ECV (37 ± 4 vs. 33 ± 5%, p = 0.002), supporting previous reports of greater extent of diffuse fibrosis affecting multiple organs in SSc patients with diffuse cutaneous involvement [7]. However, such a small difference in native T1 and ECV values may not permit diagnosis in individual patients. Other CMR tissue characteristics showed no significant difference between the two groups.

**Table 4:** Tissue characterisation in diffuse SSc vs. limited cutaneous SSc

	**Diffuse SSc**	**Limited SSc**	**P value**
	**(n = 10)**	**(n = 9)**	
Volume fraction of LGE > 2SD (%)	4 (1-6)	4 (2-5)	0.981
Volume fraction of oedema (T2 STIR SI >1.9, %)	12 (2-17)	14 (0-21)	0.939
Volume fraction of T1 > 990 ms (%)	63 (47-78)	45 925-68)	<0.001
Precontrast T1 (ms)	1011 ± 24	1002 ± 32	0.010
Postcontrast T1 (ms)*	440 ± 44	466 ± 53	0.367
λ	0.63 ± 0.19	0.49 ± 0.22	0.032
ECV (%)	37 ± 4	33 ± 5	0.002

Data are mean ± SD unless otherwise indicated.

*At 20 minutes post gadolinium administration.

λ, Myocardial partition coefficient (Lambda); *ECV*, Extracellular myocardial volume; *LGE*, Late gadolinium enhancement; *STIR*, Short Tau inversion recovery.

For postcontrast T1, λ and ECV, the number of SSc patients included in the analysis is 14 rather than 19.

### Correlation of myocardial T1 and ECV to LV function and SSc disease severity

**Figure 3:**
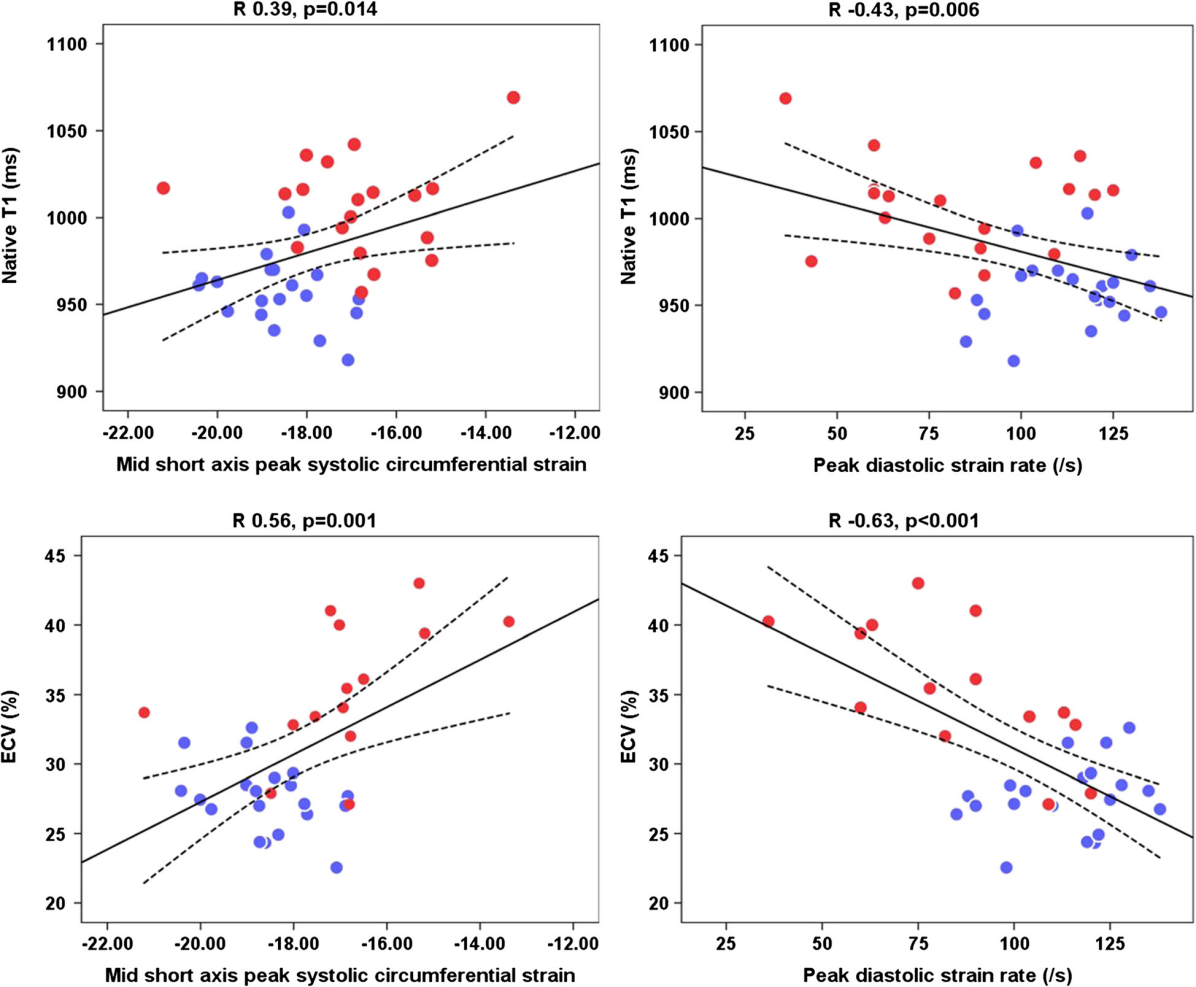
**Correlation of myocardial T1 and ECV to peak circumferential systolic strain and peak diastolic circumferential strain rate.** ECV, extracellular volume. Red dot indicates systemic sclerosis. Blue dot indicates control.

There was significant, moderate correlation of myocardial involvement detected by T1 and ECV to indices of SSc disease activity and severity: ECV and mRSS (R 0.60, p = 0.03), ECV and SSc VDAI (R_S_ 0.60, p = 0.04); native T1 and mRSS (R 0.55, p = 0.02), native T1 and SSc VDAI (R_S_ 0.55, p = 0.05), native T1 and serum CRP (R_S_ 0.41, p = 0.01) as shown in Figure 3.

The extent of myocardial oedema on T2-weighted imaging showed significant moderate correlation with peak systolic strain (R_S_ 0.49, p = 0.001) and diastolic strain rate (R_S_ -0.46, p = 0.003). Both native T1 and ECV correlated inversely with peak circumferential systolic strain (R 0.43, p = 0.006) and diastolic strain rate (R -0.46, p = 0.003; (Figure 3), suggesting that subclinical myocardial inflammation and/or diffuse myocardial fibrosis are associated with deformational abnormalities and early signs of myocardial dysfunction.

## Discussion

Our data demonstrated that subclinical myocardial changes are common in patients with SSc even with apparently normal hearts, which can be detected using multiparametric CMR. In addition to focal areas of fibrosis (as detected by LGE), there were also areas of focal myocardial oedema or inflammation (as detected by T2-weighted imaging). Further, using more sensitive techniques such as native T1 mapping and ECV quantification, we were able to demonstrate even more areas of myocardial involvement in SSc patients than conventional CMR techniques can reveal, with SSc patients showing significantly larger areas of T1 abnormality and expanded ECV, which likely represent a combination of low grade inflammation and diffuse myocardial fibrosis that are well-described disease processes in this cohort. Interestingly, T1-mapping and ECV quantification were sensitive enough to further stratify myocardial involvement in patients with diffuse SSc compared to patients with limited cutaneous SSc, with the former showing significantly larger areas of myocardial T1 abnormality and ECV expansion. Finally, T1 and ECV measures were associated with subtle myocardial systolic and diastolic dysfunction. The results of this study suggest that CMR, particularly T1 and ECV quantification, can be used for early detection of subclinical myocardial involvement in SSc patients, potentially serving as an early screening tool before overt LV dysfunction or irreversible myocardial damage occurs.

In historic autopsy studies, diffuse myocardial fibrosis has been reported as the pathological hallmark of cardiovascular involvement in SSc [7,8]. Chronic myocardial inflammation together with recurrent ischaemia-reperfusion injury and microvascular dysfunction are thought to play a crucial pathophysiological role in the development of diffuse myocardial fibrosis in SSc [4,8,9] and ultimately lead to premature cardiovascular mortality, particularly in those SSc patients with obvious clinical features of heart disease [5].

Myocardial fibrosis is characterised by excessive deposition of extracellular matrix proteins, rich in collagen [31], which ultimately affects myocardial structure and function, and is associated with impaired systolic and diastolic function, cardiac chamber dilation and arrhythmias [32,33]. LGE CMR has been traditionally used to image regional fibrosis/scarring in the myocardium, based on the distribution difference of gadolinium in between healthy and diseased myocardium [15]. The LGE technique has significant limitations in the assessment of diffuse myocardial fibrosis, where the entire myocardium may be affected more homogeneously, as occurs with SSc, where there may be little unaffected myocardium.

Native (precontrast) T1 and ECV mapping are novel CMR markers that provide a quantitative measure of tissue characterisation without relying on relative signal comparisons [15]. T1 maps are quantitative, pixel-wise representations of the T1 relaxation time of the underlying myocardial tissue [16]. Increase in native T1 values is non-specific and can be seen in acute myocardial oedema, infarction, myocarditis, amyloidosis and diffuse fibrosis [14,17,34,35]. Postcontrast T1 is dependent on renal function, body fat distribution, dose of contrast used, the time delay in measurement after contrast administration, but calculating the ECV may minimise these confounding effects by incorporating the pre- and post-contrast myocardial and blood T1, partition coefficient (λ), and adjusting for the haematocrit [36]. In the absence of oedema or other causes of ECV expansion such as amyloid and prior myocardial infarction, ECV increases are considered the best non-invasive surrogate of diffuse fibrosis [36]. ECV has been validated against histological collagen proportion [37], and correlates with poor early cardiovascular outcomes [38]. In this study, postcontrast T1 values were found to be poor measures of diffuse fibrosis compared to native T1 and ECV calculation. Due to the greater variability of this measurement, postcontrast T1 has not been considered as a preferred method of assessment of diffuse myocardial fibrosis in the consensus statement on Myocardial T1 mapping and extracellular volume quantification by the Society for Cardiovascular Magnetic Resonance (SCMR) and CMR Working Group of the European Society of Cardiology [36].

We showed increased native T1 and expanded ECV in SSc patients without cardiovascular symptoms. It is difficult to separate how much of the increase in T1 was due to myocardial inflammation as opposed to diffuse myocardial fibrosis, as both would increase native T1 values (and hence also ECV). There was a degree of myocardial oedema as shown by results using T2-weighted imaging, but the areas of oedema detected are not directly subtractable from areas of abnormality detected by T1, since these are different techniques with different sensitivities and specificities for oedema [14]. Native T1 is significantly more sensitive to myocardial water than conventional T2-weighted imaging, so areas of oedema detected by T1 are expected to be larger than those by T2-weighted imaging, even in just a single disease process. In this cohort, both myocardial inflammation and diffuse fibrosis likely co-exist, and thus, CMR findings (as for any other diagnostic imaging modality) must be interpreted within the clinical context. Trying to distinguish between myocardial inflammation and diffuse fibrosis based on imaging alone may be challenging; the chronicity and relapsing nature of this disease must be taken into account, which can result in active myocardial inflammation over existing diffuse fibrosis from a previous episode. This is an inherent limitation that lends a necessary uncertainty, but currently no non-invasive diagnostic test can achieve this goal and no other cardiac imaging modality can provide more information on myocardial tissue characteristics than multiparametric CMR at this time. In either case, this study shows that CMR can detect subclinical myocardial involvement in SSc patients whose hearts would appear otherwise normal using conventional measures; longitudinal studies following disease course and trials assessing response to treatment strategies may shed more light onto the clinical meaning of T1 and ECV abnormalities in this cohort.

Despite the absence of global functional impairment, we found impaired peak circumferential LV systolic strain in SSc patients, in keeping with previous reports [39]. There was also evidence of diastolic dysfunction with impaired peak diastolic strain rate, elevated E/E’ and increased left atrial dimensions. Although there were no major clinical effects on the SSc patients in this study, the presence of low grade myocardial inflammation, T1 abnormalities and ECV expansion may not be benign [16,31-33] and may ultimately lead to focal or diffuse fibrosis. Certainly, an autopsy study has previously found focal and interstitial myocardial fibrosis in SSc patients who had sustained high prevalence of ventricular arrhythmias and conduction disturbances, intractable congestive cardiac failure and sudden cardiac death [8].

T1 and ECV measures both correlated inversely with peak systolic strain and peak diastolic strain rate in SSc patients. Several reports (including assessment of focal myocardial fibrosis by LGE) have confirmed that myocardial fibrosis precedes strain abnormalities and that fibrosis is a significant contributor to the pathogenesis of myocardial relaxation abnormalities [40,41]. Notably, in hypertensive patients, diastolic function improves after treatment with aldosterone antagonists, likely reflecting an antifibrotic effect of these drugs [42]. In diabetes, cardiac dysfunction, relating to loss of contractile reserve and abnormal myocardial stiffness, is proportional to the degree of extracellular matrix deposition [43]. Our findings support the hypothesis that in SSc, adverse myocardial processes may lead to diffuse myocardial fibrosis and are associated with impairment in myocardial strain.

We have found that both ECV and native T1 are useful for the assessment of myocardial involvement in SSc. In patients who are unable to tolerate gadolinium, native T1 may be used as a surrogate biomarker for myocardial fibrosis [15,17], if other causes of increased T1 are unlikely. In this study, ECV and native T1 both correlated positively with indices of SSc activity and severity, indicating that CMR may be useful in assessing both myocardial disease severity and activity in SSc. Furthermore, ECV and native T1 are increased even in SSc patients without focal fibrosis on LGE imaging, suggesting that these novel CMR markers provide additional information on tissue characterisation beyond that achieved by LGE.

### Limitations

Our study has several limitations. First, the number of SSc patients included in this study is small; nevertheless the control group was well chosen and large significant differences in the parameters measured have been observed. Second, native myocardial T1 values may increase with myocardial oedema, myocardial infarction, myocarditis or amyloidosis [17,34,35] and are not specific for myocardial fibrosis as discussed. Third, T2 mapping was not performed in this cohort, which would have been an interesting comparison; however, T2 mapping at the present time seems to have a large inter-individual variability [43-45], which may not have helped significantly in distinguishing between myocardial water from inflammation versus that in an expanded extracellular space. Fourth, besides T1 mapping there is no other serum or histological test performed to support the presence of diffuse myocardial fibrosis in our patients; in this study of early disease in asymptomatic patients, no myocardial biopsy for histological correlations could be justified. Finally, we used the VDAI as a measure of disease activity in SSc. However, several other scores do exist which could have been used, which all have their own limitations.

## Conclusions

In conclusion, subclinical myocardial involvement is common in SSc patients without cardiac symptoms, as measured by T2-weighted imaging, native T1, quantitative LGE and ECV measurement; and likely signifies a combination of myocardial inflammation and diffuse fibrosis which correlated with both SSc disease activity and skin fibrosis severity, as well as with subclinical impairment of systolic and diastolic strain despite the preserved LV ejection fraction. These findings support a mechanistic role for myocardial inflammation and possible diffuse fibrosis in preclinical as well as advanced SSc disease. The results of this study highlight key advantages of multiparametric CMR to track these preclinical changes, which may be useful in the clinical setting and possibly as surrogate endpoints for therapeutic trials. Finally, our data suggest that native T1 and ECV measurement may add incremental information to LGE focal fibrosis quantification, and that focal and diffuse fibrosis may reflect different underlying pathological processes.

## Abbreviations

CMR: Cardiovascular magnetic resonance; CVD: Cardiovascular disease; ECG: Electrocardiogram; ECV: Myocardial extracellular volume; ESR: Erythrocyte sedimentation rate; LGE: Late gadolinium enhancement; LVEF: Left ventricular ejection fraction; mRSS: Modified Rodnan skin score; SSc: Systemic sclerosis; SI: Signal intensity; ShMOLLI: Shortened Modified Look-Locker Inversion Recovery; SSFP: Steady-state free precession; STIR: Short-Tau inversion recovery.

## Competing interests

Competing financial interests.

We have no financial conflicts of interests to declare.

Competing non-financial interests.

US patent pending 61/387,591: SKP, MDR. SYSTEMS AND METHODS FOR SHORTENED LOOK LOCKER INVERSION RECOVERY (Sh-MOLLI) CARDIAC GATED MAPPING OF T1. September 29, 2010. All rights sold exclusively to Siemens Medical.

US patent pending 61/689,067: SKP, MDR. COLOR MAP DESIGN METHOD FOR IMMEDIATE ASSESSMENT OF THE DEVIATION FROM ESTABLISHED NORMAL POPULATION STATISTICS AND ITS APPLICATION TO CARDIOVASCULAR T1 MAPPING IMAGES.

All other authors have reported that they have no relationships relevant to the contents of this paper to disclose.

## Authors’ contributions

NABN, PMM, BPW, SN and TDK were involved in the study conception and design. NABN, JMF and TDK were involved in data acquisition. NABN, SKP, VMF, JMF, ABSR and TDK were involved in data analysis. NABN, SPK, VMF, SN and TDK were involved in data interpretation. All authors read and approved the final draft.
